# Evaluation of Continuing Professional Development Program for Family Physicians 

**Published:** 2013-04

**Authors:** Syed Irfan Karim, Farhana Irfan, Riaz Qureshi, Naghma Naeem, Eiad Abdel Mohsen Alfaris

**Affiliations:** 1 Dr. Syed Irfan Karim, MBBS, MD(USA), MCPS, MRCGP(UK), Assistant Professor & Consultant Family Medicine, Deputy Director Family Medicine Residency Training, Department of Family and Community Medicine. King Khalid University Hospital, College of Medicine, King Saud University, Saudi Arabia.; 2 Dr. Farhana Irfan, MBBS, MCPS, MRCGP(UK), PGCertMedEd (UK), Assistant Professor, King Saud University Chair for Medical Education Research and Development, Department of Family and Community Medicine. King Khalid University Hospital, College of Medicine, King Saud University, Saudi Arabia.; 3 Prof. Riaz Qureshi, MBBS, FRCGP(UK), Distinguished Professor, Department of Family and Community Medicine. King Khalid University Hospital, College of Medicine, King Saud University, Saudi Arabia.; 4 Dr. Naghma Naeem, MBBS, MMEd (UK), Lecturer, King Saud University Chair for Medical Education Research and Development, Department of Family and Community Medicine. King Khalid University Hospital, College of Medicine, King Saud University, Saudi Arabia.; 5 Eiad Abdel Mohsen Alfaris, MBBS, MSc, MRCGP, MMed, Professor of Family Medicine, Specialist in Medical Education, Department of Family and Community Medicine. King Khalid University Hospital, College of Medicine, King Saud University, Saudi Arabia.

**Keywords:** Family Physician, Continuing medical education, Continuing professional development, Program evaluation, CRISIS

## Abstract

***Objectives: ***To evaluate the King Saud University Continuing Professional Development (CPD) Program for Family Physicians in relation to the Convenience, Relevance, Individualization, Self-Assessment, Interest, Speculation and Systematic (CRISIS) criteria.

***Methodology:*** A descriptive study was conducted at King Saud University (KSU) in Riyadh, Saudi Arabia. The authors used the six strategies of Convenience, Relevance, Individualization, Self-Assessment, Interest, Speculation and Systematic (CRISIS) for evaluation. The program was independently analyzed by the three authors using CRISIS framework. The results were synthesized. The suggestions were discussed and agreed upon and documented.

***Results:*** The results indicate that KSU-CPD program meets the CRISIS criteria for effective continuing professional development and offers a useful approach to learning. The course content covers specific areas of practice, but some shortcomings were found that need to be improved like self assessment area and individual learning needs analysis.

***Conclusion:*** This program is suitable for Family Physicians, as it is well planned and utilizes most of the principles of CRISIS, but there is still room for improvement. Designing a program for general practitioners using hybrid model that offers a blend of e-learning as well as face-to-face learning opportunities would be an ideal solution.

## INTRODUCTION

The dynamic nature of health care necessitates that physicians remain competent and up to date through Continuing Professional Development (CPD). The challenge is to develop effective CPD Programs. CRISIS, an acronym (convenience, relevance, individualization, self-assessment, interest, speculation and systematic), is a practical tool for developing and evaluating CPD programs. Lifelong learning is an essential goal of education as a means to improve the quality of life for an individual, culture or a society.

As professionals, physicians are obliged to remain current about advances and trends in medicine and health care delivery. A medical qualification, provides the knowledge and skills necessary to enter the profession, this is by no means the final step of the educational process for physicians. The constant changes in health care have resulted in the need for physicians to continually seek educational opportunities in order to maintain their competence. This is achieved through participation in a variety of educational activities. Continuing medical education (CME) is not a new concept; its need has been well documented and is now widely accepted.^1^

Many countries are now moving from a ‘knowledge and skills base’ CME system, towards a system that seeks to promote the wide-ranging competencies needed to practice high quality medicine that Continuing professional development (CPD) entails.^2^ Continuing professional development extends beyond traditional CME. In CPD, principles of adult learning are followed.^3^ It encompasses a range of learning activities through which professionals not only develop, but also maintains their knowledge and skills throughout their careers. In addition to traditional educational themes, it covers subjects like doctor-patient communication, interdisciplinary team skills and risk management, broadly termed as ‘Skills for the New Millennium’.^4^

Although CPD and CME can be, and are frequently used interchangeably, a great majority of literature has now defined CME as being an ingredient of CPD.^5^ As one academic has put it, ‘CPD is a process that includes continuing medical education (CME) with regards to medical knowledge and skills in addition to subjects such as leadership, communication skills, and whatever skills are needed to be a competent physician.^6^

Evaluation is an essential part of the educational process. The focus of evaluation is quality improvement. All medical organizations require evaluation as part of their quality assurance procedures; the challenge has been how to make it effective. To maintain, encourage and evaluate quality in all forms of CME, a set of educational criteria are available. Harden & Laidlaw (1992) have introduced criteria with the acronym CRISIS, as a means of both developing and evaluating CPD.^7^ The application of the CRISIS criteria to a CME program will highlight any areas needing improvement. CRISIS model has been applied widely and proved to be effective in various areas.^1^ Features of the CRISIS criteria are given in Table-I. Many studies have evaluated the effectiveness of CME/ CPD based on participant satisfaction^8^, but locally, little has been done for its evaluation, particularly using a set of criteria like CRISIS. This study aims to evaluate the effectiveness of a Family Physicians’ CPD Program in relation to these criteria.

**Table-I:** Crisis Model

Convenience-makes voluntary participation easy
Relevance-reflects the user’s day today role and requirements in medical practice
Individualization- to suit the individual needs
Self assessment for self remedy
Interests-to arouse attention and encourage learner participation
Speculation and-to recognize grey areas

**Fig.1:**
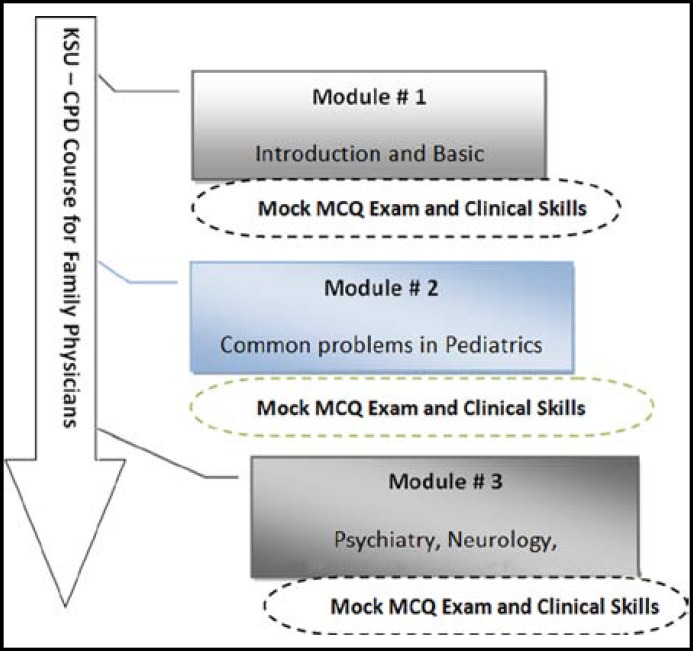
The modular approach

***Context: ***Kingdom of Saudi Arabia (KSA) is committed to providing quality health care for all. Training of health professionals is an essential prerequisite for achieving this goal. In KSA, longitudinal CPD courses for health professionals are scarce.

***Description of the CPD Program: ***The King Saud University (KSU) CPD program for Family Physicians was initiated in KSA, Riyadh in 2009, under the umbrella of Family and Community Medicine Department of College of Medicine, KSU in collaboration with KSU Chair for Medical Education Research and Development. This is an interactive CME/CPD program specifically designed for Family Physicians. The program is in modular format and provides a wide range of timely and challenging topics relevant to the practice of Family Medicine. It covers areas like, Internal Medicine, Obstetrics and Gynecology, Surgery, Pediatrics, Emergency Medicine, Psychiatry, Ophthalmology, Orthopedics, Dermatology, ENT and Radiology. The overall goal of the program is to augment physician knowledge, enhance competence and performance in practice, and improve patient outcomes. The content is updated regularly to retain educational integrity. Different types of instructional methods are used in the program including interactive lectures with scenarios and clinical and communication skills development sessions. Pre and post test and mock exams including written and OSCEs are used for formative assessment and feedback.

## METHODOLOGY

The CRISIS model consists of six strategies which are described in Table-I. The authors used the six strategies namely convenience, relevance, individualization, self assessment, interest and speculation for evaluation. The program was independently analyzed by the three authors using CRISIS framework. The results were synthesized. The suggestions were discussed, agreed upon and documented.

## RESULTS

***Convenience:*** Provide “just-in-time” learning to suit the user.

***Place:*** King Khalid University Hospital, King Saud University, Riyadh was selected as an ideal place as this is accessible and one of most popular University Hospital known by almost all Physicians.

***Time:*** The half day / week course on Thursdays was started as most of the Family Physicians (FPs) are relatively free on this day to attend a course.

***Pace:*** The program is divided into a series of modules in which the whole course is given in a series of smaller sessions over a longer period (usually several weeks), so that the doctors can choose the ones they find most useful and skip any sessions in between. This choice was given considering it may improve convenience for some.

***Relevance*** *:* “Content used in the day-to-day role of the learner”.

Topics covered in CME program are relevant, seen as being of practical importance and dealing with everyday problems. They not only cover theoretical aspects but also other areas of competence required for medical practice in which the family physicians face difficulty. Relevance of the topics is identified, using the wise men approach to cover common and important or serious illnesses amenable to medical care and conditions for which management methods have improved. The content was developed collaboratively by the university academics and family doctors/physicians.

***Individualization:*** “Just-for-you” learning.

Individuals are given the choice of the subject matter they wish to learn. The needs of the doctors are addressed by keeping into consideration the amount of time they are willing to spend and the preferred learning strategies. The participants are FP’s, either working in an urban or some rural setting. The individual learning needs of participants is identified by their feedback. A course prospectus has been designed to show the contents of the course and schedule. The participants can choose the modules most relevant to their need and of particular interest to them. The CME is designed for both the new FP’s as well as for the experienced ones. It consists of two parts, first addresses what every FP should know, and the second addresses the challenges related to patient management, as well as some more complex material giving the participants a holistic approach in their clinical skills.

***Self Assessment*** *:* “Assessment and feedback integrated with learning”.

Self Assessment is done by pre and post session MCQs test on the topics to be covered on that day to help participants identify gaps in their knowledge and to evaluate their learning. The self assessment consists of series of single best multiple choice questions, usually addressing the patient management problems including diagnosis and appropriate investigations and testing the application of knowledge and problem solving skills of the course participants.

The feedback is given to the learner in its simplest form of answer key. The explanation of each correct answer is given in a question and answer session towards the end, with explanation along with the references and sources for additional reading material. Approximately, every three to six months a mock MCQ/clinical skills examination is conducted on the topics that are covered by that stage, to assess their progress and evaluate their learning.

***Interest: ***A CME to be of interest must gain attention of the user. A few examples of the session in this program are: “Terminal care in Family practice”, “Approach to a patient with hypertension”, etc.

These CME sessions are relevant to the needs of patient care. Family Physicians (FP) is usually the first person to be contacted by the patient, so all FP’s attending these sessions get the benefit of learning the appropriate approach and solutions to common clinical problems. To maintain interest and motivation of participants and to cater to different learning styles different interactive instructional modalities, like presentation of scenarios in the form of cases, illustrations, videotape extracts and power point presentations are used.

***Speculation and Systematic:*** “Planned program” .

An organizing committee with members from Family Medicine Departments of Medical Institutions of Riyadh meets and finalizes the content of the program. The program is comprehensive and includes areas which are not usually covered in routine CME’s. It also focuses on areas which require special skills, such as breaking bad news, dealing with difficult patient, sensitive issues like HIV & AIDS and confidentiality. Workshops are conducted on consultation /communication skills.

The CME is announced in advance to let the participants know its coverage with a complete description of the topics covered and the time frame. Using model described by Dixon^7^, for evaluation of CME, participation is assessed using the registration data, satisfaction is assessed by evaluation forms, learning by pre and post test and performance by the ability to solve patient problems using paper case scenarios and simulated patients.

Each module feedback is taken from the participants and discussed with the team members to highlight any shortcomings and to implement a strategy to improve it in future.

## DISCUSSION

Medical knowledge continues to advance at a burgeoning pace. Maintenance of professional competence remains an exercise of lifelong learning.^9^ The issue of concern related to CME in Saudi Arabia is no longer about quantity but rather quality. Evaluating educational programs is not an easy task.^10^ Evaluation is an important part of any teaching and learning process and is the cornerstone for effectiveness and quality assurance process of CME.^11^ At the close of the 20th century, challenges facing CME are enormous. Saudi Arabia is part of this World and is facing similar challenges like any other country.^12^ Unless CME provision is based on a more secure and evaluated ground and integrated to health care delivery, many resources will continue to be an otiose with doubtful outcomes.^13^^,^^14^ One response to this challenge has been the development of programs appropriate to the needs of the practitioners. Designing good quality CPD courses appropriate to the needs of the different categories of practitioners remains a big challenge.

The result of this study indicates that the current program incorporates many learner centered elements. The curriculum of the program was developed in 2009 through a cooperative effort of the stakeholders of Family and Community Medicine and continues to be updated periodically.

 Time constraints and management are a big issue for physicians. As seen in the past, the most convenient place to learn is domestic and local.^15^ To keep pace with ***convenience ***in terms of time and place we tried to keep it central in the city and on weekend. Studying for long periods is often difficult for doctors. The modules in this program are divided and stand independently and offer spaced learning, which is believed to improve retention more than massed learning.^7^^,^^16^

Adults learn best if they see the relevance and usefulness of what they are required to learn.^17^ Relevance is basically the applicability of what is learned to clinical practice. Understanding the principles of adult education, the highest priority should be given to procedural knowledge (“knowing how”) rather than declarative knowledge (“knowing that”)^18^ and to frequent, important illnesses amenable to care, conditions for which management methods have recently improved and where education can improve previously poor management.^19^^,^^20^

Considering, the fact that, most physicians list the ***relevance*** of the topics as the most important factor to attend an educational program^21^^,^^22^, this program offers sessions with practical application and dealing with everyday problems rather than just those of academic interest.^21^^,^^22^ The program can be further improved by taking the individual learning needs into account, which implies that the organizers should consider some sort of needs analysis of the participants.

***Individualization*** is clearly linked to relevance. Educational interventions are more likely to be successful if they are modeled and catered towards individual learning needs and preferences, and focused on the learning component of education.^23^ Individuals attending the CPD course have different educational and professional backgrounds, therefore, their needs differ. To cope with their requirements, the program has different modules, a page layout is designed to help the learners find parts of the program that are of particular interest to them and match their needs.

***Self-assessment*** is an ideal way of identifying learning needs and assessing whether these have been addressed by the program. A feature that distinguishes a successful program from an unsuccessful program is the incorporation of a self assessment component.^10^ It encourages physicians to evaluate their competencies and understanding of the topic, and to remedy any gaps identified. Self-assessment is, however, not always valid and involves biases which are both conscious (self-deception) and unconscious (inadequate self-knowledge).^24^

A formative assessment approach is used to measure the progress of learners towards reducing or eliminating the gap. In the current program, pre and post test performance scores and Mock Examinations scores are being used for formative assessment and feedback. However, actual practice in workplace is not being assessed.

As mentioned by Knowles^3^, Andragogy is based on how adults learn and their attitude towards it and motivation for learning. Traditional lectures are the least preferred method of any educational program.^25^ It is now time to shift to the new constructivist model where learners participate actively. This program has incorporated varied learning methods like clinical cases for knowledge application, considering that using multiple methods of instruction is better, of greater use and more ***interesting ***for the participants.^26^^,^^27^ It is likely to enhance participant’s activity and a possibility to bring a change in professional attitude and practice.^28^ Incorporating an independent study module in future will enhance course credibility.

In the real world, family practice is full of grey areas where physicians have to deal with controversial issues. Usually, the educational programs concentrate on aspects which are established facts and ignore areas where there is controversy or no single correct answer.^7^ The curriculum in this program was designed considering the fact that it should be ***systematic ***emphasize on selected topics and recognize grey areas like dealing with angry patient, breaking bad news, HIV etc. Confronting such issues in an educational activity helps the doctor to tackle them in day to day practice. All issues can be discussed openly in a safe and secure environment.

The last of the CRISIS dimensions is ***speculation***. One of the neglected areas in medical education is undoubtedly organized continuing educational programs for the general practitioners. It should address the real needs of the physicians. For evaluating the effectiveness of the program, organizers evaluate the post session feedback of attendees. The feedback is used to determine the educational needs and whether the learner’s objectives were met during the session or not.^29^ This is a promising program, as it is planned and utilizes the principles for continuing education.

The curriculum evaluation on CRISIS criteria found that the program fulfilled the CRISIS criteria in a number of ways. However, needs assessment is missing and self assessment requires improvement.

## CONCLUSION

In conclusion, this program scores considerably well on all the dimensions of the CRISIS criteria, however, needs assessment of participants is missing and improvement in workplace practice needs to be assessed. Designing a program for general practitioners using hybrid model that offers a blend of e-learning as well as face-to-face learning opportunities would be an ideal solution. We recommend the CRISIS criteria for others developing continuing professional development courses in future as organized educational programs are a significant need that must be met, if we are to continue the advancement of physician learning.
